# Street-level noise in an urban setting: assessment and contribution to personal exposure

**DOI:** 10.1186/s12940-015-0006-y

**Published:** 2015-02-28

**Authors:** Tara P McAlexander, Robyn RM Gershon, Richard L Neitzel

**Affiliations:** Department of Environmental Health Sciences, Johns Hopkins Bloomberg School of Public Health, 615 N Wolfe St #5041, Baltimore, 21205 MD USA; Philip R Lee Institute for Health Policy Studies, School of Medicine, University of California San Francisco, 3333 California Street, Ste 280, San Francisco, 94118 CA USA; Department of Environmental Health Sciences and Risk Science Center, University of Michigan, 1415 Washington Heights, 6611 SPH I, Ann Arbor, 48109, MI USA

**Keywords:** Street-level noise, Urban soundscape, Urban noise exposure

## Abstract

**Background:**

The urban soundscape, which represents the totality of noise in the urban setting, is formed from a wide range of sources. One of the most ubiquitous and least studied of these is street-level (i.e., sidewalk) noise. Mainly associated with vehicular traffic, street level noise is hard to ignore and hard to escape. It is also potentially dangerous, as excessive noise from any source is an important risk factor for adverse health effects. This study was conducted to better characterize the urban soundscape and the role of street level noise on overall personal noise exposure in an urban setting.

**Methods:**

Street-level noise measures were obtained at 99 street sites located throughout New York City (NYC), along with data on time, location, and sources of environmental noise. The relationship between street-level noise measures and potential predictors of noise was analyzed using linear and logistic regression models, and geospatial modeling was used to evaluate spatial trends in noise. Daily durations of street-level activities (time spent standing, sitting, walking and running on streets) were estimated via survey from a sample of NYC community members recruited at NYC street fairs. Street-level noise measurements were then combined with daily exposure durations for each member of the sample to estimate exposure to street noise, as well as exposure to other sources of noise.

**Results:**

The mean street noise level was 73.4 dBA, with substantial spatial variation (range 55.8-95.0 dBA). Density of vehicular (road) traffic was significantly associated with excessive street level noise levels. Exposure duration data for street-level noise and other common sources of noise were collected from 1894 NYC community members. Based on individual street-level exposure estimates, and in consideration of all other sources of noise exposure in an urban population, we estimated that street noise exposure contributes approximately 4% to an average individual’s annual noise dose.

**Conclusions:**

Street-level noise exposure is a potentially important source of overall noise exposure, and the reduction of environmental sources of excessive street- level noise should be a priority for public health and urban planning.

**Electronic supplementary material:**

The online version of this article (doi:10.1186/s12940-015-0006-y) contains supplementary material, which is available to authorized users.

## Background

The complex urban soundscape is shaped by a combination of environmental noise from transit systems, road traffic, construction, industry, the built environment, population density and other sources. These sources are additive to any other sources of noise that individuals may be exposed to, the most important of which has historically been the workplace. Certain occupations (e.g., manufacturing, construction, etc.) present well documented risk of excessive noise exposure, and even today, after over 40 years of occupational noise regulation in the United States (US), there are still estimated to be as many as 30 million US workers exposed to excessive occupational noise levels annually [1]. However, while occupational sources of noise are well characterized, the contribution of other sources, and that of the urban soundscape (e.g., the totality of noise in the urban setting), to an individual’s total noise exposure has not been well described. This is important because excessive noise, resulting from any combination of sources, is known to cause adverse health effects [2]. For example, noise from road traffic and other sources has been associated with increased risk of hypertension [3,4] and coronary heart disease [5,6], psychological stress and annoyance [7-10] and sleep disturbance in adults [11,12]. In children, cognitive impacts and increased psychological stress from noise exposure have been documented [13-16]. The US Environmental Protection Agency (EPA) has set a 24-hour (daily) exposure limit for noise of 70 dBA, and chronic exposure above this daily limit is believed to be sufficient to cause noise-induced hearing loss (NIHL) [17]. Even below this level, noise has been linked to health problems [18]. Given the health implications and the continuing increase in urbanization globally, there is a need to better understand the sources of urban noise and the risk of exposure to hazardous levels of noise among urban dwellers.

Perhaps the most important contributor to the urban soundscape is street-level sound. Given the ubiquitous exposure to street-level sounds among urban dwellers, and the potential for annoyance and health effects from this noise, strict urban noise control measures for street-level sources of noise are increasingly being implemented. However, even in cities such as New York City (NYC), which has rigorous regulations on, and enforcement of, nuisance noise sources (e.g., loud radios and car alarms) and construction–related noise, there is little focus on street-level noise (e.g., noise from roadway traffic, commercial activities, etc.,), though some studies have evaluated noise from mass transit in NYC [19,20]. The extent and magnitude of levels of street noise and air pollutants in NYC has recently been assessed [21], but the methodology used did not measure personal exposures, but rather levels at 10 ft above street level, well above the elevation of the heads of pedestrians. Variation in street-level noise with regards to vehicle traffic and road proximity has been explored, [18] but most studies on traffic noise have relied on modeling of noise levels from a network of roads, land use regression, or extrapolation of models based on a small number of noise samples [22-24]. While these are cost-effective approaches to estimate traffic noise levels, they can neglect factors in the urban built and natural environment that may mitigate or exacerbate exposure to street noise levels, including temporal changes in noise, built environment factors, and vulnerable areas and individuals. These other factors have particular relevance for understanding exposure to street-level noise in the urban environment.

We conducted this study to address gaps in our knowledge of the urban soundscape. The goals of the study were threefold: first, to measure street-level noise across NYC and to evaluate potential correlates of street-level noise; second, to estimate the public’s exposure to street-level noise by combining these measured noise levels with previously-collected street level exposure duration data from nearly two thousand individuals who lived or worked in NYC; and third, to estimate the contribution of street level noise exposure to total noise exposure in an urban population. We hypothesized that the NYC borough of Manhattan would present the greatest risk of exposure to higher street noise levels due to its high density and relatively greater levels of pedestrian and traffic activity, compared to other city boroughs.

## Methods

All study protocols were approved by the Columbia University Institutional Review Board (CUMC IRB-AAAE2243 and CUMC IRB-AAAD1614).

### Street noise measurements

We measured street-level noise in the summer of 2010 at a large number of locations, and across different times of day, to assess spatial and temporal variations in noise levels. Extensive quality control measures were implemented to ensure reliability of noise level data collection. Sixty sites in Manhattan were chosen to reflect regions of low, medium and high street level noise based on the number of noise complaints per square acre made via the noise complaint hotline maintained by the NYC city government. (Sarah Williams, e-mail communication, 2009). We included for sampling several particularly heavily trafficked areas (e.g., Columbus Circle and Times Square), as well as four small urban parks (referred to as “pocket parks”). Additionally, we selected sites in the Bronx, Queens, and Brooklyn (30 total sites in these boroughs) based on noise complaints in these boroughs. Five additional sites were chosen to reflect regions of noise complaints in the less-populated borough of Staten Island, for a total of 35 locations in the four outer (e.g., non-Manhattan) boroughs. Noise levels were measured during early morning rush hour (7:00–9:30 AM), late morning (9:30 AM-12:00 PM), early afternoon (12:00–2:30 PM), and late afternoon rush hour (2:30–5:00 PM).

Noise measurements occurred only on weekdays (Monday-Friday) to avoid expected differences in noise levels and activity patterns between weekdays and weekends. The duration of each measurement was 10 minutes. Measurements were made using a Q-300 dosimeter (Quest Technologies, a 3 M Corporation, Oconomowoc WI) worn by research staff, with the dosimeter microphone positioned mid-shoulder. Dosimeters were calibrated at the beginning and end of each measurement day to insure accuracy. The equivalent continuous average noise exposure level (L_EQ_) and the maximum noise level (L_MAX_) in A-weighted decibels (dBA) were recorded for each measurement. The L_EQ_ represents the average noise level received over a period of time that typically spans minutes to hours, while the L_MAX_ represents the very highest exposure received over a period of seconds or even milliseconds. The dosimeters were configured according to the recommendations of the US Environmental Protection Agency (3 dB time-intensity exchange rate, 75 dBA criterion level, 8 hour criterion time, slow response, no measurement threshold) [17]. We used a 40–110 dBA measurement range to avoid the potential bias which would result from excluding noise levels below the typical measurement range of 70–140 dBA.

During each measurement, research staff recorded time and geographic location as well as additional relevant information on nearby conditions (e.g., ambulance passing by, dog barking, etc.). Research staff also noted the vehicular traffic nearby, including the number of moving vehicles. This crude traffic count was then used to classify sites as high, medium, and low traffic volume for analysis purposes.

### Data analysis

R 64 (R Project, freeware) was used for data cleaning and analysis; statistical tests were considered significant where p < 0.05. Descriptive statistics were calculated on measured L_EQ_ and L_MAX_ noise levels overall and by borough, time of day, traffic level, and nearby conditions. We used a linear mixed effects model to estimate the within- and between-measurement location variance. A linear mixed effects model was constructed to predict L_EQ_ by borough, traffic level, and time of day. Measurement location was treated as a random effect and borough, time of day, traffic level, and nearby conditions were treated as fixed effects. We chose this model over other models with spatial components, such as a spatial lag model or geographic weighted regression, due to the non-random selection of sample locations.

A logistic mixed effects model was developed to predict average noise levels ≥80 dBA, for which the EPA recommended daily exposure duration is about 2.5 hours. This model was also developed using the Lmer (mixed effects modeling) function and was fit using adaptive Gaussian Hermite approximation to produce log-likelihoods of effect estimates. Measurement location was treated as a random effect, and fixed effects were traffic level, time of day, and a dichotomized borough category (Manhattan vs. other boroughs). We dichotomized borough because there were more samples in Manhattan than any other individual borough and because Manhattan is denser and busier than the outer boroughs.

### Geospatial mapping

Geographic coordinates were generated for each site sampled using ArcGIS software. The nearest address and, when appropriate, nearest intersection, were used to identify the site’s location. Inverse-distance weighted interpolation utilized the nearest neighbor method to interpolate noise level values, considering the nearest 3–5 data points. This generated a map of the noise data and interpolated estimates, which was then overlaid on top of a map of NYC, provided by the U.S. Census Bureau [25]. Note, the map contours are not meant to be predictive, and estimated levels from a smoothed map were not included in our analyses and thus the map generated is meant to merely serve as a visual guide.

### Personal noise exposure assessment

#### Survey data collection

A previously-described survey [26,27] was used to assess the frequency and duration of common noisy activities among the NYC public in 2009. The anonymous survey was distributed to individuals who lived or worked in NYC using a street-intercept methodology at street fairs. The survey evaluated exposure to five sources of noise: occupational noise, non-occupational noise (concerts, sporting events, etc.,) transit noise, listening to music, and time spent standing, walking, and running on NYC streets, as well as use of hearing protection during these activities.

We created exposure estimates for the sampled individuals using an approach we have described previously [26,28,29]. Briefly, we used survey responses to estimate the total annual duration of exposure to each of the five noise sources: occupational activities, non-occupational activities, transit use, listening to music through headphones or stereos, and NYC streets. We subtracted the sum of the annual durations of four of these five sources (excluding listening to music, which was not mutually exclusive with the other categories) from the total number of hours (8760) in a year period, and assigned the remaining duration to the sixth category, home and other miscellaneous activities. This approach yielded durations of exposure for all six sources of noise for each individual subject.

### Noise exposure assignment

We assigned transit noise levels based on our previous assessment of NYC transit noise [19]. Occupational, non-occupational, music, and home and other miscellaneous activities were each assigned noise levels derived from a matrix of mean exposures derived from peer-reviewed publications [26] (Additional file 1: Table S1). Street-level exposures were assigned using the average street noise level we measured in the NYC borough in which subjects lived, or, for non-NYC residents, the borough in which they worked. We believe the mean activity-specific levels assigned through this approach are reasonably accurate, though application of a mean noise level does not accurately capture the distribution of exposures among individuals in the sample. Subjects who reported “always” or “almost always” using hearing protection during exposure to specific noise sources received a 10 dBA reduction in the noise level assigned to those specific activities [26].

### Data analysis

We estimated noise exposures to individual subjects for each exposure source using the following formulas, described in detail elsewhere [26].

1 $$ {L}_{eq,n{(8760)}_i}=10{ \log}_{10}\frac{1}{8760}\left({t}_{ni}{10}^{L_n/10}\right) $$

where (*L*_*EQ, n(8760)i*_) is the annual noise exposure, in dBA, to each source *n* for individual *i*, with exposure duration *t* to level *L*, normalized to a period of 8760 hours. This normalization allows for direct comparison of exposures to sources that have different exposure durations. We used Equation 1 to assign noise exposures for occupational activities, non-occupational activities, music, home and other miscellaneous sources, and street time. A slightly modified form of equation 1 was used to estimate noise exposures to transit; this modified equation differentiated between the time and noise level of periods spent waiting for transit from those spent riding transit.

Total annual exposures (*L*_*EQ, TO(8760)i*_) were computed by logarithmically averaging the annual exposure levels and durations for each of the six sources. The fraction *F* of total exposure due to each of the *n* sources was then computed for each individual *i* using equation 2.

2 $$ {F}_{ni}=\frac{10^{L_{n(8760)i}}}{10^{L_{TO(8760)i}}} $$

To evaluate the relative importance of street noise exposures to total noise among the sampled individuals, we repeated the above analyses but excluded the estimated street-level noise exposure for each subject. We then compared this estimate to the estimate that included all six noise sources.

## Results

### Street-level noise measurements

A total of 329 valid noise measurements were collected (Table 1). The p-values displayed for the Borough categories represent the comparison between Manhattan and the average noise levels for all outer boroughs combined. Significant differences in L_EQ_ noise levels between categories were present for all measured variables. The majority of noise measurements were ≥70 dBA, but levels varied by borough, time of day, traffic level, and nearby conditions. Ten of the 16 pocket park measures were also ≥70 dBA, with three measures greater than 75 dBA. Specific nearby conditions associated with the highest street noise levels included sirens, high levels of pedestrian traffic, the presence of large water fountains, and construction activity. Measurements obtained when sirens were sounding (emergency or security vehicles) and when construction activities were actively ongoing were likely to exceed 80 dBA. Forty-six percent of the total variance in measured street L_EQ_ levels was due to within-site variability (data not shown), suggesting that variations in noise levels over the course of a day were roughly equivalent to differences between site. The results of the mixed effects linear regression analysis are displayed in Table 2a. The factors most predictive of high L_EQ_ levels were location in Manhattan, high traffic level, and evening time period.

**Table 1:** **Descriptive statistics for measured L**_**EQ**_ **noise levels**

**Variable**	**Category**	**N**	**Mean L**_**EQ**_ **(dBA)**	**Std. Dev.**	p-value*	**N (%) of measurements ≥ 70 dBA**	**N (%) of measurements ≥ 80 dBA**
Total		329	73.4	6.1		241 (73.3)	46 (14.0)
Borough	Manhattan	259	74.6	5.4	<0.0001	214 (82.6)	41 (15.8)
	Outer boroughs	70	69.1	6.6		27 (38.6)	5 (7.1)
	Bronx	20	67.5	6.1		6 (30.0)	1 (5.0)
	Brooklyn	20	68.8	6.0		6 (30.0)	1 (5.0)
	Queens	20	70.5	7.3		11 (55.0)	2 (10.0)
	Staten island	10	70.5	7.4		4 (40.0)	1 (10.0)
Time of day	Early morning	69	74.3	4.2	0.0007	59 (85.5)	7 (10.1)
	Late morning	100	72.4	6.4		64 (64.0)	13 (13.0)
	Afternoon	99	72.4	6.5		65 (65.7)	9 (9.1)
	Evening	61	76.0	6.0		53 (86.9)	17 (27.9)
Traffic level	Low	93	68.4	4.8	<.0001	35 (37.6)	1 (1.1)
	Medium	120	73.8	4.3		98 (81.7)	12 (10.0)
	High	116	77.2	5.9		108 (93.1)	33 (28.4)
Nearby	Vehicle traffic	188	74.6	5.4	<.0001	153 (81.4)	31 (16.5)
conditions	Pedestrian traffic	28	75.4	7.0		23 (82.1)	4 (14.3)
	Music	6	72.8	3.2		5 (83.3)	0 (0.0)
	Construction	21	73.0	7.0		15 (71.4)	4 (19.0)
	Sirens	18	76.8	4.3		17 (94.4)	4 (22.2)
	Subway	10	71.5	7.0		5 (50.0)	1 (10.0)
	Quiet	51	67.6	5.3		17 (33.3)	2 (3.9)
	Water (fountain)	7	74.2	4.1		6 (85.7)	0 (0.0)

*Oneway ANOVA.

**Table 2:** **Mixed effects regression models**

**Variable**	**Category**	**Estimated L**_**EQ**_ **noise level (dBA)**	**95% CI (dBA)**
a. Linear regression			
(Intercept)		64.3	61.7, 67.0
Borough	Brooklyn	1.7	−1.6, 5.1
	Manhattan*	6.0	3.4, 8.5
	Queens*	4.5	1.1, 7.8
	Staten Island*	4.9	0.8, 9.1
Time of day	Late morning	−0.5	−1.7, 0.8
	Afternoon	−0.9	−2.3, 0.3
	Evening	1.0	−0.4, 2.3
Traffic level	Medium*	4.0	2.63, 5.4
	High *	7.7	6.3, 9.1
		Odds ratio	95% CI
b. Logistic regression, odds of noise level ≥80 dBA			
(Intercept)**		0.0028	0.00004, 0.085
Borough	Manhattan	1.5	0.036, 6.5
	Afternoon	0.6	0.018, 2.3
	Evening*	0.5	0.01, 11.1
Traffic Level	Medium	a	a
	High**	a	a

*Indicates significant coefficient or OR, p < 0.05.

**Indicates significant coefficient or OR, p < 0.01.

a Indicates instability in the estimate due to small numbers.

Table 2b shows the results of our mixed effects logistic regression analysis, which explored the odds of L_EQ_ noise level exceeding 80 dBA by borough, traffic level, and time of day. Measurements made during late morning had slightly elevated odds of L_EQ_ ≥80 dBA, while measurements made in medium or high traffic conditions had substantially increased odds, though these odds were not robust (e.g., had very wide confidence intervals, reflecting instability in the regression model).

**Figure 1:**
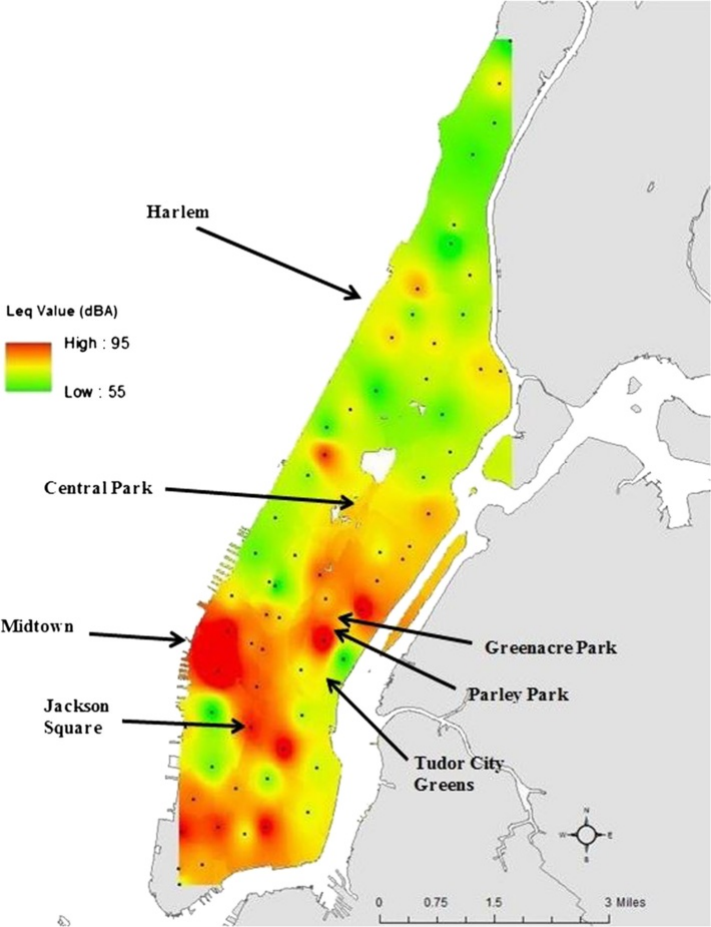
**Estimated noise levels across Manhattan.** Indicates measurement location.

**Figure 2:**
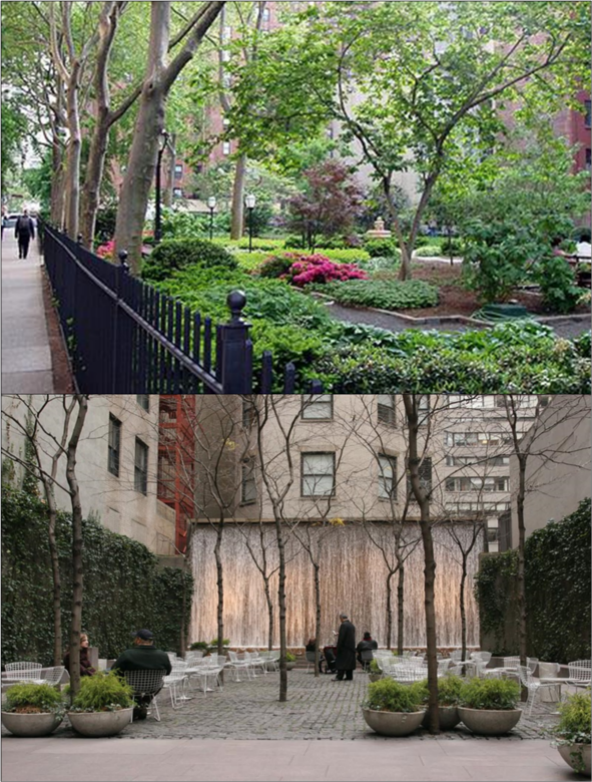
**Image of “pocket parks”.** These parks are located throughout Manhattan, particularly in the Midtown business district. The top image is of Tudor City Greens, and the image below is Paley Park.

Figure 1 displays the estimated noise levels across Manhattan, including several pocket parks sampled within the borough. Estimates were restricted to only Manhattan due to small sample sizes in the outer boroughs. Noise levels were generally highest in lower Manhattan. Figure 2 displays images of pocket parks in Manhattan. While pocket parks are desirable features in an urban environment, and represent a potential refuge from noise exposure, the noise levels presented in Table 3 suggests that these parks are in fact associated with moderate to high levels of noise, and that there is relatively small variation between parks for a given time of day.

**Table 3:** **Pocket park L**_**EQ**_ **noise levels, recorded at four times throughout the day**

**“Pocket park”**	**Early morning L**_**EQ**_ **(dBA)**	**Late morning L**_**EQ**_ **(dBA)**	**Early afternoon L**_**EQ**_ **(dBA)**	**Late afternoon L**_**EQ**_ **(dBA)**
Paley park	76.4	78.9	74.4	73.3
Greenacre park	77.9	72.4	73.9	73.7
Tudor city greens	70.4	65.9	66.8	70.4
Jackson square	70	69	66.6	67.1

### Personal noise exposures

Survey data on street-level noise exposures were collected as part of a larger study of noise exposures in NYC. A total of 1894 subjects completed surveys that addressed time spent on NYC streets as part of our street fair sampling methodology. The majority of subjects were from Manhattan. In the sample of subjects, 52.6% were male, 97.5% were transit users, 96.1% lived in NYC, and 85.8% worked in NYC. The distributions of age, occupation, and durations of exposure to each of the noise sources we evaluated were compared to NYC census data and found to be similar. While our sampling procedure was not designed to ensure a representative sample for the NYC area, we believe these results indicate that our sample is reasonably representative of NYC mass transit users. However, the generalizability of our results to individuals living in other cities may be limited. Overall, subjects reported spending 81.9 ± 88.8 minutes on NYC streets each day, with individuals in the Bronx having the greatest mean daily duration (83.2 min) and individuals on Staten Island having the lowest (58.1 min). The amount of time spent on NYC streets did not vary significantly between individual boroughs or between Manhattan and the outer boroughs (oneway ANOVA, p > 0.05). The average annual amount of time spent on NYC streets across all subjects was 498.5 ± 540.2 hours (data not shown), or roughly 6% of the total hours in a year.

Noise exposure estimates for the sample are displayed in Table 4. More than 90% of participants exceeded the EPA’s 70 dBA recommended exposure limit when exposure from street noise was included in each subjects’ total exposure estimates and use of hearing protection devices was considered. The mean estimated total exposure across all subjects including street noise increased slightly but significantly (p < 0.05) compared to estimates that did not include street noise (76.4 ± 5.0 without vs. 77.0 ± 5.0 dBA with street noise). When street noise was considered, it contributed slightly more than 4% of the total exposure on average. All exposure sources except home-related sources contributed more to total exposure than street noise. The greatest contribution of exposure, regardless of whether or not street noise was considered, was listening to music, which contributed roughly 46% of total noise exposure, on average. Non-occupational and occupational activities each contributed roughly 25% of total noise exposure. Personal exposures were significantly higher in Manhattan than in the outer boroughs (80.5 ± 5.4 in Manhattan vs 74.2 ± 4.5 dBA in the outer boroughs, data not shown).

**Table 4:** **Annual noise exposures by source with and without consideration of street noise**

					**Percent of total noise exposure**			
		Annual HPD*-adjusted L_**EQ8760**_ **with street noise**			**Without street exposure**		**With street exposure**	
**Noise exposure source**	**N subjects**	**Mean**	**SD**	**% > 70 dBA**	**Mean**	**SD**	**Mean**	**SD**
Total	1894	77.0	5.0	90.5	100.0	--	100.0	--
Street	1894	58.2	5.3	2.3	--	--	4.1	8.0
Home	1894	56.7	1.0	0.0	2.9	7.4	2.2	5.0
Transit	1847	65.0	4.5	10.0	14.7	20.3	13.6	18.2
Occupational	1631	66.4	7.2	22.7	17.1	25.4	16.4	24.7
Non-occupational	932	73.0	7.1	32.9	17.6	26.7	17.3	26.3
Listening to Music	1493	75.1	2.7	78.8	47.6	33.8	46.4	32.8

*HPD: use of hearing protection device.

## Discussion

Our study represents one of the first large-scale, citywide noise exposure assessments based on a large dataset of measurements. Our results indicated substantial spatial and temporal variability in street noise levels in NYC, and that street noise makes a small but potentially important contribution to the overall noise exposures of individuals who live or work in NYC. The variability in measured exposure levels may help explain the likelihood of street-level noise resulting in annoyance among those nearby; this, in turn, may have important policy ramifications for assessing and potentially regulating street-level noise. Likewise, the spatial variability observed in our measurements can be used to identify specific areas for noise abatement or traffic management interventions, or public education programs designed to reduce exposures. Our results supported our hypothesis that, among the five NYC boroughs, Manhattan would have the highest street noise levels and greatest personal noise exposures, likely due to its density and higher levels of traffic activity. Our measurement results also generally agreed with the noise complaint volume for our selected sampling locations; in other words, locations with high noise complaints generally had higher measured street noise levels. However, complaint volume and measured noise level were not perfectly correlated, reinforcing the notion that there are other aspects beyond just intensity of exposure that affect annoyance and perhaps other health effects of noise. These aspects may include source of noise, temporal predictability of noise, and the degree to which affected individuals can control their own exposure.

A majority of measured locations had noise levels ≥70 dBA, which has significant implications for human health, as risk of hearing loss and other non-auditory health effects grows with increasing exposure. Measured noise levels were especially high in areas with high levels of reported traffic, during evening and early morning time periods (commuting times), and in Manhattan. Some of the highest noise measurements occurred in the presence of sirens, construction, or heavy pedestrian traffic; all of these activities are known sources of community annoyance. Surprisingly, even pocket parks, which have the potential to represent quiet areas within which urban denizens can seek refuge from noise, were found to have moderate to high levels of noise exposure. Some of these parks, and particularly those in the most densely-populated areas of Manhattan, had higher measured noise levels than surrounding streets and public areas. Given the emerging importance to human health of time spent in quiet environments [10], in combination with the relationship between higher noise levels and lower perceived quality of life and health, this suggests that greater attention should be paid to park siting and design.

In addition to annoyance, our recently published estimates of predicted noise-induced hearing loss among our overall sample of individuals in NYC [27] suggest that nearly all of those studied are at risk of a small NIHL from their daily exposures. While the current study did not assess health outcomes, it provides valuable information on noise levels at locations across NYC, and suggests that efforts to reduce street (vehicular) noise levels in NYC are warranted to protect the public’s health.

Our linear mixed effects regression model identified several significant predictors of noise levels measured in this study. Specifically, noise levels were found to increase with subjectively-rated traffic volumes. These results should be interpreted with caution given the small sample size for the outer boroughs, but the results are consistent with our hypothesis that Manhattan would likely have higher noise levels than other boroughs, as vehicular traffic is so high. Similar high levels associated with vehicular traffic density have also been reported by Zannin et al. [30]. A study of environmental noise in Nigeria found the highest levels during the morning rush hour, underscoring the assumption that traffic is a large contributor to environmental noise [31]. This is a plausible assumption, given the fact that in both linear and logistic regression, traffic level was a significant predictor of elevated noise levels. This is consistent with findings from a 2002 survey of roadside traffic noise in Hong Kong, where To et al. found that traffic volume was the most significant factor relating to urban noise [22]. Other sources of noise at the street level may contribute, most notably construction and loud and voluble conversations, but our data clearly indicate that vehicular traffic was the most significant contributor.

Our population-based exposure estimates suggest that street-level noise exposure contributes 4% of total noise exposure for the NYC public, and that excessive noise levels are ubiquitous throughout NYC. Our findings indicate that it is difficult to avoid potentially harmful noise exposure in NYC, and that even time spent in pocket parks can potentially represent a risk to hearing health, and perhaps to other health outcomes.

Our study had a number of limitations. First, some potential sources of noise were likely not accurately assessed. Our use of mean levels derived from the published literature for exposure to noise from occupational, non-occupational, music, and home and other miscellaneous activities likely underestimates the individual variability in total noise exposure estimates; however, no other mechanism exists with which to create these estimates. In particular, in-home and nighttime noise exposure, which may be especially important from an annoyance and disturbance perspective, are important but poorly-characterized aspects of noise exposure. While our estimates of in-home exposure were based on published exposures measured in homes in urban areas, it is possible that in-home exposures in NYC are higher than other areas due to factors such as population density, housing stock quality, and street traffic volumes. Similarly, since exposure to environmental hazards is a function of an individual’s daily activities and patterns, we have likely missed important variability in the durations of exposure to specific noise sources. Our measures of exposure durations for time spent in various activities rely completely on self-report, and time spent on streets or in noisy areas may have been subject to recall bias. This bias may be especially important relative to other sources, as the amount of time spent on streets is much smaller than the amount of time subjects were exposed to the other five sources of noise we considered. It is also possible that the source of our sample – adults surveyed at a street fair – may reduce the generalizability of our estimates of time spent on streets, as individuals who attend street fairs may spend more time on streets than those who do not. Data related to firearms noise among individuals who fire weapons for recreational or occupational purposes were not collected; while these exposures are expected to affect a small fraction of our sample, for those individuals who do use firearms this is likely their dominant exposure. If weekend noise levels in NYC differ substantially from weekday levels, our estimates of street noise exposures are biased high, and the true contribution of street noise to total noise exposure is lower than reported here. Finally, our noise level data were based on only short-term (10-minute) measurements of daytime noise, however, Gan et al. noted that short-term noise samples are highly correlated with noise models and are therefore an acceptable method for assessing exposure to noise [32]. Additional, longer-term, multi-day measurements are clearly needed to fully characterize the NYC environment.

This study underscores the need for additional research in this area. For example, long-term personal noise exposure monitoring could be used to validate our exposure estimates, and would also likely identify additional exposure sources we have not already considered. Additional studies should consider random sampling of noise levels in urban settings, and large-scale audiometric testing could also be used to evaluate the actual extent of hearing loss in urban populations; such data are currently not available in the US.

## Conclusions

Based on our results and the existing literature on noise-related health effects, street-level noise in NYC has the potential to cause auditory and non-auditory health effects [33]. Our study has several policy implications and considerations. Since we have shown that it is difficult to escape noise exposure from NYC streets, it is reasonable to consider whether noise levels should be a consideration in the design of urban parks and inhabited structures to promote places of respite from noise exposure. Additional focus on noise exposures in parks and structures could result in the use of construction materials with greater noise attenuation, incorporation of different spatial elements in parks to reduce noise from nearby activities, and other factors. Furthermore, since the findings from this study and from several other studies identify traffic as a major source of noise pollution, transit policies and projects should consider noise as a top priority [22,34]. Since excessive noise appears unavoidable from some urban environments such as NYC and may be associated with a range of adverse health effects, it may be appropriate for noise exposure assessment to be added as a requirement for environmental impact statements and health impact assessments [35]. This is particularly true when potential urban noise refuges, including pocket parks, are being designed and developed. These and other preventive steps may help reduce the risk of excessive exposure and likely result in substantial public health benefits.

## Additional file

Additional file 1: Table S1. L_EQ_ noise levels assigned based on review of peer-reviewed literature.

## Abbreviations

dBA: A-weighted decibels
EPA: Environmental Protection Agency
L_EQ_: Equivalent continuous average noise exposure level
NIHL: Noise induced hearing loss
NYC: New York City
US: United States
