# Supplementary material for: Street-level noise in an urban setting: assessment and contribution to personal exposure
Source: Environ Health. 2015 Feb 28;14:18. doi: 10.1186/s12940-015-0006-y (PMC4350859; doi:10.1186/s12940-015-0006-y)
Supplement: Additional file 1: Table S1. — LEQ noise levels assigned based on review of peer-reviewed literature. [file 12940_2015_6_MOESM1_ESM.doc]

**SUPPLEMENTAL INFORMATION**

Street-level noise in an urban setting: Assessment and contribution to personal exposure

Tara P. McAlexander^1^, Robyn R. M. Gershon^2^, Richard L. Neitzel^3*^

^1^Department of Environmental Health Sciences

Johns Hopkins Bloomberg School of Public Health

615 N Wolfe St #5041, Baltimore, MD 21205

^2^Philip R Lee Institute for Health Policy Studies

School of Medicine

University of California San Francisco

3333 California Street, Ste 280, San Francisco, CA 94118

***Corresponding Author:**

^3^Richard L. Neitzel

Department of Environmental Health Sciences and Risk Science Center

University of Michigan

1415 Washington Heights, 6611 SPH I Ann Arbor, MI 48109

**Email addresses**

TPM: [tmcalex1@jhu.edu](mailto:tmcalex1@jhu.edu); RRMG: [robyn.gershon@ucsf.edu](mailto:robyn.gershon@ucsf.edu); RLN: [rneitzel@umich.edu](mailto:rneitzel@umich.edu)

1 Table, 1 page

Table S1. L_EQ_ noise levels assigned based on review of peer-reviewed literature

Adapted from Table S1, Neitzel RL, Gershon RR, McAlexander TP, Magda LA, Pearson JM. Exposures to transit and other sources of noise among New York City residents. *Environ Sci Technol*. 2012 Jan 3;46(1):500-8.

| **Source/Industry** | **Assigned L_EQ_** |
| --- | --- |
| Transit |  |
| Rail |  |
| Platform | 78.2 |
| Vehicle | 80.1 |
| Bus |  |
| Platform | 75.3 |
| Vehicle | 76 |
| Ferry |  |
| Platform | 77.7 |
| Vehicle | 72.9 |
|  |  |
| Occupational |  |
| Agriculture | 89.8 |
| Construction | 88.1 |
| Education/Research | 79.3 |
| Entertainment | 88.0 |
| Food services | 79.7 |
| Healthcare | 70.7 |
| Landscaping | 86.6 |
| Maintenance | 82.0 |
| Manufacturing | 89.1 |
| Military | 90.5 |
| Professional | 71.9 |
| Retail | 78.1 |
| Transportation | 83.8 |
| Utility | 82.6 |
| Homemaker, retired, or unemployed | 70 |
|  |  |
| Nonoccupational |  |
| Play in band | 97.4 |
| Concerts | 96.0 |
| Sporting events | 92.6 |
| Power tools | 90.8 |
| Motorcycle | 100.0 |
|  |  |
| MP3 players & stereos | 86.2 |
|  |  |
| Home/other miscellaneous activities | 58.2 |

All values are mean L_EQ_s (or midpoints, where means were not reported)
